# Prevention of oxaliplatin-related neurotoxicity by ω-3 PUFAs: A double-blind randomized study of patients receiving oxaliplatin combined with capecitabine for colon cancer

**DOI:** 10.1097/MD.0000000000023564

**Published:** 2020-12-11

**Authors:** Xinjie Zhang, Haitao Chen, Yi Lu, Chao Xu, Wang Yao, Lu Xu, Runzhe Zhang, Liping Zhang, Qinghua Yao

**Affiliations:** a Zhejiang Chinese Medical University; b Department of Nutrition; c Department of Integrated Traditional Chinese and Western Medicine, the Cancer Hospital of the University of Chinese Academy of Sciences (Zhejiang Cancer Hospital); d The First Affiliated Hospital of Zhejiang Chinese Medical University; e Key Laboratory of Traditional Chinese Medicine Oncology, the Cancer Hospital of the University of Chinese Academy of Sciences (Zhejiang Cancer Hospital), Hangzhou, Zhejiang, China.

**Keywords:** ω-3 polyunsaturated fatty acids, chemotherapy, colon cancer, oxaliplatin-related neurotoxicity, quality of life

## Abstract

**Background:**

Peripheral neurotoxicity (PN) is a frequent side effect of oxaliplatin treatment, and also is its dose-limiting toxicity. Studies have confirmed that ω-3 polyunsaturated fatty acids (ω-3 PUFAs) had a neuroprotective effect. However, the efficacy of ω-3 PUFAs on the prevention of oxaliplatin-related neurotoxicity remains unclear. We assessed the effect of ω-3 PUFAs on the neurotoxicity in colon cancer patients treated by oxaliplatin combined with capecitabine.

**Methods:**

In a randomized, double-blind, placebo-controlled study, 179 patients with colon cancer receiving oxaliplatin combined with capecitabine were recruited, and randomly assigned to take ω-3 PUFAs, 640 mg t.i.d during chemotherapy and 1 month after the end of the treatment or placebo. All patients were treated with chemotherapy for 6 treatment cycles. The incidence and severity of PN were evaluated, and the nerve conduction was measured before the onset of chemotherapy and 1 month after treatment. In addition, the quality of life was also accessed using Chinese version of European organization for research and treatment of cancer quality of life questionnaire.

**Results:**

The incidence of PN in the ω-3 PUFAs group and placebo group was 52.22% and 69.66%, respectively (*P* = .017). In addition, there was a significant difference in the severity of PN between the 2 groups (*P* = .017). In terms of motor and sensory nerve conduction, the sensory action potentials amplitude of sural nerve in the ω-3 PUFAs group and placebo group after chemotherapy treatment were (15.01 ± 3.14) and (13.00 ± 3.63) μ V respectively, suggesting there was a significant difference in the 2 groups (*P* = .000). In addition, the mean score of the global health-status/quality of life was obviously higher in the ω-3 PUFAs group than that in the placebo group.

**Conclusion:**

ω-3 PUFAs seem to reduce the incidence and severity of oxaliplatin-related neurotoxicity, and improve the quality of patients’ life, indicating it is expected to be a potential drug for the treatment of oxaliplatin-related neurotoxicity.

## 1 Introduction

Global cancer statistics have reported that over 1.8 million new colorectal cancer (CRC) cases and 881,000 deaths were estimated to occur in 2018, and the incidence and mortality of CRC was 6.1% and 9.2% in all cancer cases, respectively.^[1]^ Therefore, it is an important to effectively control the development of CRC. Chemotherapy is a standard adjuvant treatment of CRC.^[2]^ Oxaliplatin, a third generation platinum-based agent, is the principal chemotherapeutic drug for the treatment of CRC. Although oxaliplatin can significantly improve the patients’ overall survival,^[3]^ the peripheral neurotoxicity (PN) caused by oxaliplatin is a frequent dose-limiting toxicity.^[4,5]^ Some degree of PN occurs in nearly all patients with receiving oxaliplatin.^[4]^ The mechanism of oxaliplatin induced neurotoxicity is unclear. Several studies have suggested that the occurrence of PN was related to the accumulation of oxaliplatin in the dorsal root ganglia.^[6,7]^ In clinic, calcium and magnesium infusions are often used to treat oxaliplatin-related neurotoxicity,^[8,9]^ but the curative effect does not well, and the most effective way is to suspend to use oxaliplatin.

ω-3 polyunsaturated fatty acids (ω-3 PUFAs) are essential fatty acids in the human body, including eicosatetraenoic acid (EPA), and docosahexenoic acid (DHA). Accumulated studies have shown that EPA and DHA were often used in the treatment of neurodegenerative diseases by exerting anti-oxidative, anti-inflammatory, and neurotrophic effects.^[10,11]^ Moreover, DHA had an prevention role in treating peripheral neuropathy caused by bortezomib in patients with multiple myeloma.^[12]^ However, little is known about the effect of ω-3 PUFAs on the treatment of oxaliplatin-related neurotoxicity. Herein, we had conducted a randomized, double-blind, placebo-controlled clinical trail to evaluate the efficacy of the ω-3 PUFAs on the treatment of neurotoxicity in patients receiving oxaliplatin combined with capecitabine.

## 2 Patients and methods

### 2.1 Patients

The protocol of this study was approved by the Medical Ethics Committee of Zhejiang Cancer Hospital (IRB-2015-216). Before the study was conducted, written informed consents of patients were obtained.

**Figure 1:**
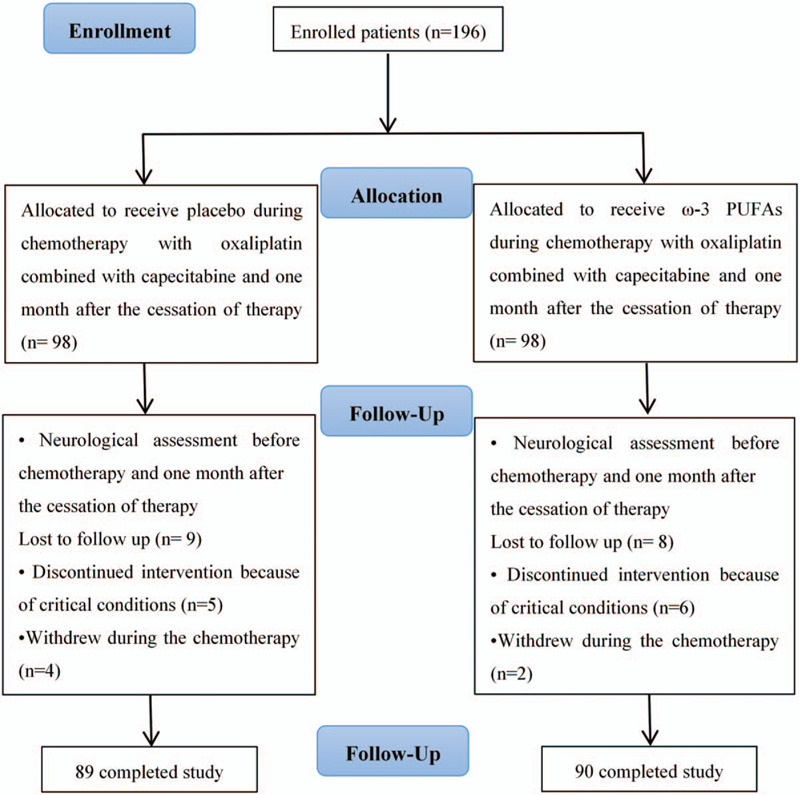
Diagram of randomization of patients with colon cancer to receive ω-3 polyunsaturated fatty acids or placebo.

In this randomized, double-blind, placebo-controlled study, participants were recruited for the study from the department of integrated traditional Chinese and western medicine of Zhejiang Cancer Hospital from January 2017 to December 2019. The inclusion criteria will consist of: patients with stage III and IV colon cancer if they have to begin receiving oxaliplatin combined with capecitabine; ECOG sore ranged from 0 to 1; not having autoimmune disease, diabetes, or renal, hepatic, cardiac, and parathyroid disorders; not having peripheral neuropathy disease; not receiving any other chemotherapeutic drugs; not taking neuroprotective drugs, such as calcium and magnesium infusions; not taking ω-3 and vitamin/mineral supplements and other nutrition medications; not allergic to fish and fish products; and without history of having other cancers. Exclusion criteria will consist of: changes chemotherapy regimen; affected by any acute disease during the study; refuses to continue chemotherapy; unwilling to continue the study; also, patients who were less than 90% compliant with treatment will be excluded. Figure 1 shows a summary of the study design.

### 2.2 Randomization and blinding

The randomization assignment will be performed using opaque envelopes with the numbers of the experiment groups. The participants who meet the criteria will be randomly allocated into 2 groups:

1) 640 mg ω-3 PUFAs (54% DHA, 10% EPA) 3 times a day during chemotherapy and 1 month after the end of therapy or
2) placebo capsules, that were equal calorie and similar in appearance to ω-3 PUFAs capsules, and similarly administered. Noteworthy, the dose of ω-3 PUFAs was determined based on previous clinical study.^[13]^ All capsules were produced from Zhejiang hailisheng Co., Ltd, China. For the regimen of chemotherapy: all patients received oxaliplatin 130 mg/m^2^ combined with capecitabine 1000 mg/m^2^ every 3 weeks for 6 cycles.

For blinding, a person not involved in the study will make the randomization list, assigning participants to the ω-3 PUFAs or placebo group. ω-3 PUFAs and placebo tablets will be placed into unlabeled identical capsules. All participants and investigators will be blinded to the random assignments.

### 2.3 Measurements

Before the beginning of the intervention, a questionnaire about patients’ cancer history, medications, diseases, and probable supplement use will be recorded. According to previous study,^[13]^ the neurotoxicity was measured at 1 month after the end of chemotherapy according to the classification criteria of neurotoxicity by the same neurologist.^[14]^ Nerve conduction studies were conducted unilaterally (right side) by using a Nicolet/VIASYS Viking Quest EMG Machine based on the standard methods. The distal motor latency, peak to baseline amplitude of compound muscle action potentia, and motor cond uction velocity of the nerves in tibial, peroneal, and ulnar were measured as the motor nerve conduction assessment. The sensory action potential amplitude (a-SAP) and sensory conduction velocity were measured for peroneal, and ulnar nerves as the sensory nerve conduction measurements.

Besides, the quality of life was accessed using Chinese version of European organization for research and treatment of cancer quality of life questionnaire (EORTC QLQ-C30 questionnaire).^[15]^ The EORTC QLQ-C30 questionnaire has 30 items arranged into 9 scales and 6 single items. The scales are divided into 5 function scales (physical, role, cognitive, emotional, and social functions); 3 symptom scales (fatigue, nausea, or vomiting, pain) and 1 global health-status/quality of life scale. The 6 single items address specific symptoms: dyspnoea, insomnia, appetite loss, constipation, diarrhoea, and financial problems. The questionnaire was officially translated into the Chinese language. The higher score of function scales and global health-status, the better the patient's quality of life; and the higher score of symptom scales, the worse the patient's quality of life. The questionnaire was officially translated into the Chinese language.

### 2.4 Statistical analysis

All date was presented as mean ± S.D. All analyses will be performed with IBM SPSS Statistics 19 (IBM Corp, Armonk, NY). The 2 groups of patients’ baseline characteristics were statistically evaluated using Chi-squared or Fisher's test. Analysis of covariance (ANCOVA) test was used to determine the differences of nerve conduction measurements between the 2 groups at the end of study, adjusting for baseline values and covariates. The difference of PN incidence and the severity of PN between the 2 study groups was estimated by using logistic regression and ordinal regression analysis, respectiely. *P* < .05 was considered statistically significant.

## 3 Results

### 3.1 Patient characteristics

196 patients were enrolled in the study and randomly assigned to receive ω-3 PUFAs (n = 98) or placebo (n = 98). 11 patients discontinued the study due to a critical health conditions (including grade 4 neutropenia and hand-foot syndrome, and severe liver function damage during chemotherapy) and 6 patients were unwilling to continue during the chemotherapy (lost to follow up, n = 17). So 179 colon cancer patients completed the study, and 90 patients in the ω-3 PUFAs group and 89 patients in the placebo group (Fig. 1). The ω-3 PUFAs group and the placebo group were comparable for gender, age, performance scores, clinical stages, temperature, respiratory rates, pulse, systolic blood pressure, and diastolic blood pressure. There was no statistical significant difference between the 2 groups. Patient characteristics are displayed in Table 1.

**Table 1:** Comparison of clinical features between the 2 groups.

	Total Number (n = 179)	ω-3 PUFAs group (n = 90)	Placebo group (n = 89)	x^2^	*P*
Gender				0.005	.943
Male	93 (52.0%)	47 (52.2%)	46 (51.7%)		
Female	86 (48.0%)	43 (47.8%)	43 (48.3%)		
Age				2.011	.156
<65	85 (47.5%)	38 (42.2%)	47 (52.8%)		
≥65	94 (52.5%)	52 (57.8%)	42 (47.2%)		
ECOG score				0.137	.712
2	84 (46.9%)	41 (45.6%)	43 (48.3%)		
3	95 (53.1%)	49 (54.4%)	46 (51.7%)		
Clinical stage				0.148	.700
III	79 (44.1%)	41 (45.6%)	38 (42.7%)		
IV	100 (55.9%)	49 (54.4%)	51 (57.3%)		
BMI		18.85 ± 1.69	18.77 ± 1.52		.755
Temperature		36.74 ± 0.402	36.77 ± 0.366		.554
Respiratory rates		18.48 ± 2.833	18.38 ± 2.906		.824
Pulse (Times)		77.778 ± 9.24	78.967 ± 8.090		.361
SBP (mm Hg)		116.778 ± 15.020	116.494 ± 13.869		.896
DBP (mm Hg)		77.378 ± 9.069	77.629 ± 8.790		.851

DBP = Diastolic blood pressure, SBP = Systolic blood pressure.

### 3.2 Peripheral neuropathy

According to the classification criteria of neurotoxicity, the severity of PN in the patients of ω-3 PUFAs group was as follows: 43 patients (47.8%) did not develop PN and 47 patients (52.2%) manifested some grade of PN: 25 patients (27.8%) developed stage I of PN; 11 patients (12.2%) developed stage II of PN; 6 patients (6.7%) developed stage III of PN; and 5 patients (5.6%) developed stage IV of PN (Table 2).

**Table 2:** Oxaliplatin-induced peripheral neuropathy in the study groups.

	ω-3 PUFAs group	Placebo group	x^2^	*P*
0	43 (47.8%)	27 (30.3%)		
I	25 (27.8%)	22 (24.7%)		
II	11 (12.2%)	11 (12.4%)		
III	6 (6.7%)	17 (19.1%)		
IV	5 (5.6%)	12 (13.5%)		
No PN	43 (47.8%)	27 (30.3%)		
Number of PN	47 (52.2%)	62 (69.7%)		
Total	90 (100%)	89 (100%)		
Incidence of PN			5.716	.017
Severity of PN			11.987	.017

PN = peripheral neurotoxicity.

In placebo group, PN was not observed in 27 patients (30.3%), while 22 patients (24.7%) developed stage I of PN; 11 patients (12.4%) developed stage II of PN; and 17 patients (19.1%) developed stage III of PN. Also, stage IV of PN was seen in 12 patient (13.5%) (Table 2). These results indicated that there was a significant difference between the ω-3 PUFAs group and the placebo group in PN (*P* = .017).

### 3.3 Nerve conduction study parameters

The differences of quantitative values between 2 study groups were measured by using analysis of covariance adjusting baseline measurements. In the study, there was a significant difference in sural a-SAP between the ω-3 PUFAs group and the placebo group (*P* = .000) with a sharp decrease of sural a-SAP in the placebo group. Furthermore, the other differences of nerve conduction study parameters has not statistical significance between the 2 groups. (Tables 3 and 4).

**Table 3:** Motor nerve conduction measurements.

	Group	Pre-chemotherapy	Post-chemotherapy	*P*^∗^
Tibial nerve				
DML (ms)	ω-3 PUFAs	16.75 (1.93)	17.90 (2.33)	.000^∗^
	Placebo	16.62 (2.34)	17.29 (2.33)	.037^∗^
	*P*^#^	.646	.079	
a-CMAP (mV)	ω-3 PUFAs	11.20 (1.84)	11.63 (2.50)	.092
	Placebo	11.68 (2.49)	11.34 (2.56)	.372
	*P*^#^	.061	.447	
MCV (m/s)	ω-3 PUFAs	46.92 (5.83)	46.47 (5.70)	.595
	Placebo	46.86 (5.60)	46.59 (5.48)	.745
	*P*^#^	.939	.884	
Peroneal nerve				
DML (ms)	ω-3 PUFAs	15.61 (2.46)	15.607 (1.972)	.99
	Placebo	15.71 (2.73)	15.658 (2.25)	.881
	*P*^#^	.791	.872	
a-CMAP (mV)	ω-3 PUFAs	8.25 (2.96)	8.06 (2.83)	.658
	Placebo	7.90 (2.83)	7.88 (2.73)	.948
	*P*^#^	.42	.655	
MCV (m/s)	ω-3 PUFAs	47.61 (7.80)	46.98 (6.71)	.558
	Placebo	47.27 (6.59)	46.01 (7.77)	.245
	*P*^#^	.751	.375	
Ulnar nerve				
DML (ms)	ω-3 PUFAs	11.65 (4.46)	11.94 (4.20)	.655
	Placebo	12.27 (4.21)	12.44 (4.18)	.782
	*P*^#^	.343	.424	
a-CMAP (mV)	ω-3 PUFAs	17.18 (4.81)	17.29 (4.01)	.873
	Placebo	17.30 (4.17)	17.39 (4.08)	.886
	*P*^#^	.858	.864	
MCV (m/s)	ω-3 PUFAs	54.16 (11.11)	52.50 (10.38)	.303
	Placebo	52.78 (12.31)	52.32 (11.27)	.667
	*P*^#^	.434	.909	

a-CMAP = amplitude of compound muscle action potential, DML = distal motor latency, MCV = motor conduction velocity.

∗ One month after the cessation of chemotherapy.

# *P* value is reported based on the analysis of covariance.

**Table 4:** Sensory nerve conduction measurements.

	Group	Pre-chemotherapy	Post-chemotherapy^∗^	*P*-value^#^
Sural nerve				
a-SAP (μ V)	ω-3 PUFAs	14.78 (3.21)	15.01 (3.14)	.638
	Placebo	14.97 (4.08)	13.00 (3.63)	.001^∗^
	*P*^#^	.732	.000^#^	
SCV (m/s)	ω-3 PUFAs	50.89 (10.82)	50.75 (11.74)	.932
	Placebo	50.96 (9.91)	51.06 (9.58)	.942
	*P*^#^	.967	.844	
Ulnar nerve				
a-SAP (μ V)	ω-3 PUFAs	36.90 (9.31)	32.23 (9.75)	.001^∗^
	Placebo	34.76 (12.4)	29.36 (12.02)	.004^∗^
	*P*^#^	.192	.08	
SCV (m/s)	ω-3 PUFAs	52.41 (10.47)	52.10 (9.85)	.835
	Placebo	51.48 (11.64)	51.51 (10.84)	.984
	*P*^#^	.573	.707	

a-SAP = sensory action potential amplitude, SCV = sensory conduction velocity.

∗ One month after the cessation of chemotherapy.

# *P* value is reported based on the analysis of covariance.

### 3.4 Scale and item scores in the EORTC QLQ-C30 questionnaire

The scores of EORTC QLQ-C30 for all scales and items was shown in Table 5. In this study, the mean score of the global health-status/quality of life was obviously higher in the ω-3 PUFAs group than that in the placebo group (*P* = .032). For measurement of function scales, a significant reduction in physical function score was found in the placebo group than that in the ω-3 PUFAs group (*P* = .046). In addition, compared to the ω-3 PUFAs group, the mean score of appetite loss was higher in the placebo group (*P* = .025).

**Table 5:** The scores of all scales and items for Quality of Life Questionnaire-C30 in patients after chemotherapy.

	ω-3 PUFAs	Placebo	*P*
Global health status	62 ± 10.45	43 ± 12.32	.032
Physical function	48 ± 9.69	30 ± 10.39	.046
Role function	35 ± 10.35	28 ± 15.52	.124
Emotional function	40 ± 10.58	38 ± 13.25	.842
Cognitive function	56 ± 14.92	53 ± 13.44	.245
Social function	45 ± 11.42	39 ± 15.77	.675
Fatigue	52 ± 15.23	55 ± 14.78	.156
Nausea/vomiting	54 ± 18.54	63 ± 19.47	.078
Pain	62 ± 17.36	65 ± 15.25	.956
Dispnea	42 ± 9.28	48 ± 8.22	.652
Insomnia	66 ± 10.78	63 ± 7.86	.078
Appetite loss	62 ± 9.34	79 ± 14.39	.025
Constipation	42 ± 8.79	49 ± 12.71	.534
Diarrhoea	69 ± 8.67	71 ± 10.62	.658
Financial problems	68 ± 7.44	70 ± 10.33	.466

ω-3 PUFAs = ω-3 polyunsaturated fatty acids.

## 4 Discussion

Oxaliplatin is one the standard adjuvant chemotherapy drug of colon cancer, and also of the standard palliative treatment for metastases. However, peripheral sensory neurotoxicity is a common toxic effect of oxaliplatin, which is often limited the use of oxaliplatin doses and affected the efficacy of the chemotherapy treatment.^[9]^ Oxaliplatin-related PN includes acute PN and chronic PN.^[16]^ Acute PN often occurs during the treatment, which is characterized by rapid onset of paresthesia of peripheral nerves that are sensitive to cold stimuli, such as numbness or pain in extremities and oropharynx.^[17]^ Chronic peripheral neuropathy is a delayed peripheral neuropathy that occurs after repeated oxaliplatin administration, and is manifested as loss of sensation and ataxia. It was estimated that most patients who received this chemotherapeutic agent developed dose-dependent neurotoxicity.^[4]^ Therefore, oxaliplatin-related PN affected the patients’ quality of life obviously, and it is important to prevent oxaliplatin-related neurotoxicity effectively.

The aim of the current randomized, double-blind, placebo-controlled study was to confirm the efficacy of ω-3 PUFAs in prophylaxis against oxaliplatin induced PN. According to our knowledge, this is the first time to confirm the efficacy of ω-3 in reducing the incidence and severity of oxaliplatin induced PN. In the study, there was a significant difference in the incidence of oxaliplatin-related PN between the 2 groups that 47.8% of patients taking ω-3 PUFAs did not develop PN while incidence was 30.3% in the placebo group, indicating that ω-3 PUFAs had neuroprotective effects and decreased the incidence of oxaliplatin-related neurotoxicity considerably. Our results are similar to previous studies that have discovered ω-3 PUFAs reduced the incidence of PN in diabetic neuropathy.^[18]^ In addition, neurophysiologic studies improved the accuracy and precision of PN evaluation and helped to identify the severity of PN in patients with oxaliplatin. Thus, we used the classification criteria of neurotoxicity that reported previous study to identify the severity of PN in patients,^[14]^ we found that there was also a significant difference in the severity of PN between the 2 groups that ω-3 PUFAs reduced the severity of PN remarkably. These results are in accordance with previous studies that have reported the efficacy of ω-3 PUFAs in degenerative diseases. Study showed that ω-3 PUFAs could attenuate the severity of neuropathy by inhibiting the inflammatory response in patients with degenerative diseases.^[11]^ Meanwhile, a combined EPA and DHA oral administration accelerated nerve regeneration and prevented neuropathic pain though its anti-neuroinflammatory activity in mice with peripheral nerve injury.^[19]^ Also, accumulating evidence has shown that ω-3 PUFAs alleviated neuropathic pain by suppressing neuroinflammation and oxidative stress.^[20,21]^ In addition, study also reported fish oil treatment can provide some beneficial effects against cisplatin-induced peripheral neuropathy by changing latencies through anti-oxidant and anti-inflammatory, which may lead to myelinated axon regeneration.^[22]^ According to these results, we proposed that ω-3 PUFAs might prevent the incidence and severity of PN via inhibiting neuroinflammatory response.

In the current study, the nerve conduction was further analyzed, and a considerable difference was found in sural nerve a-SAP between the 2 groups that a sharp decrease in the placebo group. Similarly, Ghoreishi et al had reported that omega-3 fatty acids were protective against paclitaxel-induced peripheral neuropathy might be related to prevent the significant decrease of sural nerve a-SAP. Besides, Argyriou et al found that the decrease sural a-SAP >50% of the baseline value, as being the sole, significant predictor of worse neurological outcome in the cisplatin- and paclitaxel-based chemotherapy induced peripheral neuropathy.^[23]^ Therefore, our results indicated that ω-3 PUFAs attenuate oxaliplatin induced PN may be also associated with remission of sural nerve a-SAP reduction.

Numerous studies have confirmed that chemotherapy-induced PN affected patient's quality of life, including patients’ emotional and functional domains.^[24,25]^ Therefore, based on the efficacy of ω-3 PUFAs in prevention of oxaliplatin-related PN, we further measured the patients’ quality of life. The mean scores of the global health-status/quality of life and physical function were higher after ω-3 PUFAs treatment in patients with oxaliplatin combined with capecitabine. Meanwhile, the score of appetite loss was reduced significantly in the ω-3 PUFAs group. These changes may be related to the attenuation of oxaliplatin-related PN by ω-3 PUFAs administration. Thus, these results indicated that ω-3 PUFAs improved the patients’ quality of life via alleviating PN in colon cancer patients with oxaliplatin combined with capecitabine.

However, there were some limitations in the design of this randomized, double-blind, placebo-controlled study. Lack of long-term follow-up of measurement results is a potential limitation of this study. Additionally, relatively small sample size and single-center study were another possible limitations of the current trial.

## 5 Conclusion

This double-blind, randomized study illustrated that ω-3 PUFAs may be potential neuroprotective agent for treatment of oxaliplatin-related neurotoxicity. Another double-blind, multi-center, placebo-controlled randomized clinical study is needed to confirm these results. Moreover, we will further measure the incidence and severity of PN and quality of life at 6 months or 1 year after the end of chemotherapy as indicators of long-term efficacy of ω-3 PUFAs. Besides, the mechanism of ω-3 PUFAs on the prevention of oxaliplatin-related neurotoxicity should be further studied.

## Author contributions

**Conceptualization:** Qinghua Yao, Xinjie Zhang, Haitao Chen, Liping Zhang.

**Data curation:** Xinjie Zhang, Haitao Chen, Yi Lu, Wang Yao, Runzhe Zhang.

**Formal analysis:** Xinjie Zhang, Yi Lu.

**Funding acquisition:** Qinghua Yao.

**Investigation:** Yi Lu.

**Methodology:** Haitao Chen.

**Project administration:** Qinghua Yao, Liping Zhang.

**Software:** Haitao Chen, Chao Xu, Wang Yao, Lu Xu.

**Supervision:** Chao Xu, Lu Xu, Liping Zhang.

**Writing – original draft:** Xinjie Zhang, Haitao Chen.

**Writing – review & editing:** Qinghua Yao, Yi Lu, Liping Zhang.
